# Orientation selectivity in a multi-gated organic electrochemical transistor

**DOI:** 10.1038/srep27007

**Published:** 2016-06-01

**Authors:** Paschalis Gkoupidenis, Dimitrios A. Koutsouras, Thomas Lonjaret, Jessamyn A. Fairfield, George G. Malliaras

**Affiliations:** 1 Department of Bioelectronics, Ecole Nationale Supérieure des Mines, CMP-EMSE, MOC, 13541 Gardanne, France; 2 MicroVitae Technologies, Hôtel Technologique, Europarc Sainte Victoire Bât 6, Route de Valbrillant, 13590 Meyreuil, France; 3 School of Chemistry and CRANN Institute, Trinity College Dublin, Dublin 2, Ireland

## Abstract

Neuromorphic devices offer promising computational paradigms that transcend the limitations of conventional technologies. A prominent example, inspired by the workings of the brain, is spatiotemporal information processing. Here we demonstrate orientation selectivity, a spatiotemporal processing function of the visual cortex, using a poly(3,4ethylenedioxythiophene):poly(styrene sulfonate) (PEDOT:PSS) organic electrochemical transistor with multiple gates. Spatially distributed inputs on a gate electrode array are found to correlate with the output of the transistor, leading to the ability to discriminate between different stimuli orientations. The demonstration of spatiotemporal processing in an organic electronic device paves the way for neuromorphic devices with new form factors and a facile interface with biology.

Over the past years, the field of neuromorphic devices or synaptic electronics has attracted a great deal of attention^1^. Efforts in this field have concentrated on capturing the computational efficiency of the brain by reverse-engineering it at the hardware level. In the past years, a host of neural processing functions have been demonstrated with neuroinspired devices such as short- and long- term plasticity, short- to long-term memory transition, dynamic filtering, and Spike**-**Timing**-**Dependent Plasticity, just to name a few^2^,^3^,^4^,^5^,^6^,^7^. It is well known that information processing in the brain is spatiotemporal in nature: spatial and temporal dynamics in the brain contribute to a continuum that unfolds in space and time, resulting in higher complexity neural network functionalities, for example time encoding in neural trajectories^8^. A well known paradigm of spatiotemporal processing is the orientation selectivity of cortical cells^9^. Orientation selectivity is a broadly investigated phenomenon that refers to vision. Hubel and Wiesel pioneered the understanding of visual perception through their early work on the feline visual cortex^10^,^11^,^12^. Visual cortex cells are known to change their output spiking rate selectively to light bars with differing orientation. According to Hubel and Wiesel’s theory, the receptive field of visual cortex cells has a center/surround shape (a central and a surrounding region) that consists of an excitatory (ON) and an inhibitory (OFF) region, which produce ON/OFF (ON central and OFF surrounding) or OFF/ON (OFF central and ON surrounding) center/surround structures. When these center/surround structures are superimposed into elongated forms, they produce selectivity towards a specific orientation. Although the basic model of orientation selectivity is currently well understood, there are still uncertainties about the underlying mechanisms^13^. Orientation selectivity has been recently demonstrated with neuromorphic devices and/or arrays of devices such as with memristive grids^14^, floating-gate transistor arrays^15^, and graphene oxide transistors^16^.

Another field of particular interest that is related to biology and has recently attracted a lot of interest is the field of organic bioelectronics^17^,^18^. A wide variety of organic bioelectronic devices is available for interfacing with biological systems including neural interfaces, drug delivery devices, biosensors, devices that control cell growth and enzyme-based logic devices^19^,^20^,^21^,^22^,^23^. The organic electrochemical transistor (OECT) is regarded as a benchmark in organic bioelectronics^24^,^25^. An OECT consists of an electrolyte between a gate electrode and a conducting polymer channel. The polymer channel can be gated through the electrolyte. By applying a gate voltage, ions from the electrolyte are injected to the channel and change the doping level of the conducting polymer. This leads to the modulation of the electronic current that flows through the channel. OECTs have several attractive characteristics for interfacing with biological systems including the efficient ionic-to-electronic current transduction, operation in a liquid electrolyte environment, mechanical flexibility and biocompatibility. Moreover, the channel of the OECT can be laterally gated with an electrode (i.e., the gate electrode)^26^,^27^. OECTs have also been recently proposed as neuromorphic devices^27^,^28^,^29^, and neural functions that are temporal in nature have been demonstrated in a single device level, such as short- and long-term synaptic plasticity^27^,^28^. In these devices, depending on the presynaptic timing and voltage conditions, various synaptic plasticity functions were reproduced, but without any spatial variable. Here, we demonstrate for the first time the implementation of a spatially-correlated neuromorphic function in a poly(3,4-ethylenedioxythiophene) doped with poly(styrene sulfonate) (PEDOT:PSS) based OECT. This spatially-correlated function is an analogue of orientation selectivity in a single device level. Spatially-correlated functions similar to orientation selectivity may pave the way for introducing spatial concepts in the implementation of neuromorphic functions with multi-terminal neuromorphic devices that share a common electrolyte.

## Results

**Figure 1:**
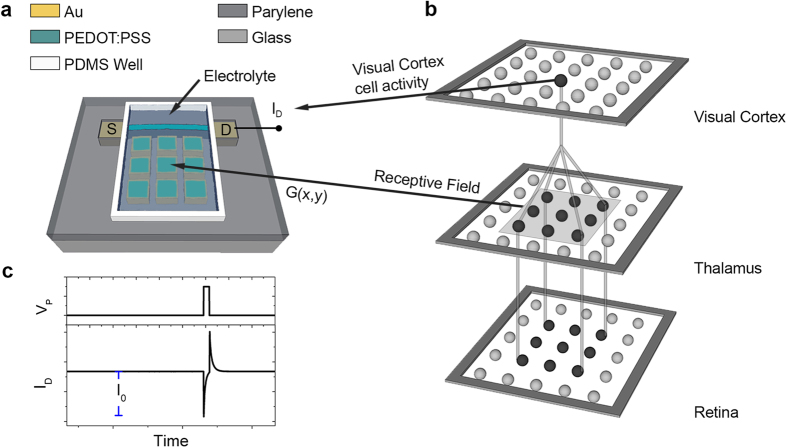
Analogy of the visual system and the multi-gated OECT. (**a**) Schematic of the multi-gate OECT device. (**b**) Simplified schematic of the visual system. The analogy of the multi-gated OECT and the thalamus/visual cortex is also depicted in Fig. 1a,b. (**c**) Measurement of the drain current *I*_*D*_ that results from a voltage pulse *V*_*P*_ at a gate electrode and the definition of drain amplitude *I*_*0*_.

The device geometry used here is depicted in Fig. 1a. The device fabrication process is described in detail in the experimental section. A glass substrate with patterned Au electrodes (source, drain electrode and an array of 3 × 3 gate electrodes) was coated with a PEDOT:PSS film in order to define a device channel with nominal dimensions of 15 mm × 0.5 mm (length × width, L × W). PEDOT:PSS was used to make the channel, as this material has been established as the standard for high performance OECTs. The device was laterally gated through a NaCl electrolyte confined in a PDMS well, with a grid of 3 × 3 gate electrodes at coordinates (x, y). For the experiments, various voltage pulses were applied at the (x, y) gate electrodes (with amplitude *V*_*P*_ and width *t*_*P*_). The resulting drain current *I*_*D*_ was simultaneously recorded and its amplitude *I*_*0*_ for t → 0 was defined in each case (see also Fig. 1c). The geometry of the multi-gated device was inspired by the visual system structure^15^,^30^,^31^. The analogy of the multi-gated OECT and the visual system is presented in Fig. 1a,b. In the visual system (Fig. 1b), optical stimuli are captured by the retina and then transmitted as optical information to the Lateral Geniculate Nucleus (LGN) cells of the thalamus via optic nerves. The information from the LGN cells is subsequently transmitted to the orientation-selective visual cortex cells in a purely electrical and/or chemical form. At that stage, the activity of a group of LGN cells (this group is also known as receptive field) is projected to a cortical cell and transformed into an orientation–dependent firing activity. In the multi-gated OECT device of Fig. 1, the (x, y) gate electrodes are regarded as the receptive field of a visual cortex cell, while the drain current *I*_*D*_ is an analogue of the activity of the cortical cell. In the multi-gated OECT, as in the case of the neural transmission from the thalamus to the cortical neurons, there is a projection from a spatial input voltage pattern of (x, y) gate inputs (that can be regarded as a vector field) into a single *I*_*0*_ output response (that can be regarded as a scalar quantity).

**Figure 2:**
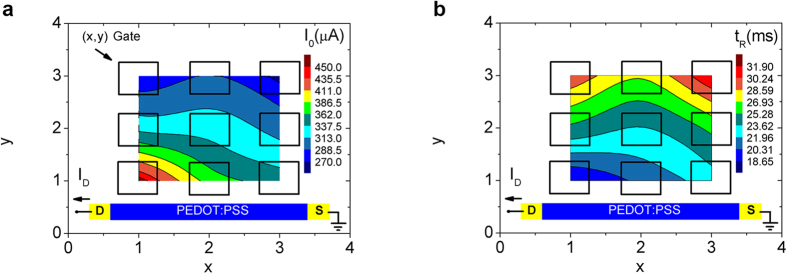
Spatial mapping of current amplitude and relaxation time. Spatial mapping of: (**a**) the resulting amplitude *I*_*0*_ of the drain current and (**b**) the relaxation time *t*_*R*_. Each (x, y) gate electrode was pulsed separately with a voltage amplitude V_P_ = 0.3 V, time width t_P_ = 50 ms and the resulting current was measured for V_DS_ = −0.2 V.

Initially a voltage pulse (V_P_ = 0.3 V, t_P_ = 50 ms) was applied at each (x, y) gate electrode and *I*_*0*_ was recorded (for source-drain voltage V_DS_ = −0.2 V). The experimental spatial *I*_*0*_ mapping of the multi-gated OECT is presented in Fig. 2a. The spatial mapping was constructed by pulsing each (x, y) gate electrode and measuring *I*_*0*_ for each case. From the spatial mapping, an increased *I*_*0*_ amplitude is obvious for the gates that are closer to the drain electrode. As described in the Supplementary Information section, from the time domain response of the equivalent circuit that describes the OECT (see also Supplementary Fig. S1 for the equivalent circuit), the amplitude *I*_*0*_ (for t → 0) can be expressed as I_0_ = V/R_E_ (equation S13 of Supplementary Information), where *V* is the input pulse amplitude and *R*_*E*_ the electrolyte resistance between the gate electrode and the PEDOT:PSS channel. Equation S13 implies that *I*_*0*_ depends on *R*_*E*_. *R*_*E*_ can be simply expressed using the equation S14, as R_E_ = ρ_E_ (d/A), where *ρ*_*Ε*_ is electrolyte resistivity, *A* is the electrode area and *d* is effective distance between the channel and the center of the (x, y) gate electrode. As shown in Supplementary Figs S2 and S3, this increased *I*_*0*_ response close to the drain electrode is attributed to the decrease of the electrolyte resistance *R*_*E*_ for smaller gate-drain electrode distances *d*. This spatial inhomogeneity in *I*_*0*_ can be used for implementing the orientation selectivity function in multi-gated OECTs.

The relaxation time *t*_*R*_ for the depolarization response of the drain current *I*_*D*_ is also calculated for pulsing every (x, y) gate using a simple exponential decay, I(t) ~ exp[−t/t_R_] and the results are presented in Fig. 2b as a spatial mapping of *t*_*R*_. Similarly with the *I*_*0*_, inhomogeneity towards the drain electrode is also evident in *t*_*R*_ mapping. For example the closest to the drain, gate electrode (x = 1, y = 1), results in the lowest *t*_*R*_ value. Assuming that polarization and depolarization responses are symmetrical (implying that the same equivalent circuit describes both processes), equation S11 of Supplementary Information gives a relaxation time that for R_E_ ≪ R_P_ can be simplified to t_R_ = R_E_∙C_P_;^32^ where *C*_*P*_ is the PEDOT:PSS capacitance. Therefore the closest gate to the drain electrode, defines the lowest *R*_*E*_, results also in the lowest relaxation time *t*_*R*_.

**Figure 3:**
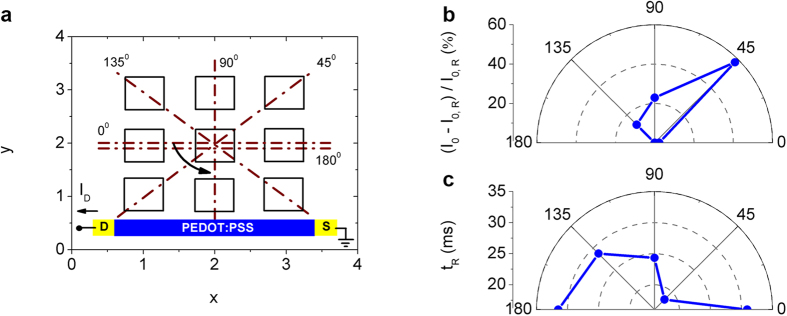
Orientation selectivity. (**a**) Definition of input spatial pulse patterns with different orientations (0°–180°) at the (x, y) gate electrodes. The spatial patterns are defined by applying pulses simultaneously at 3 gate electrodes (V_P_ = 0.3 V, t_P_ = 50 ms). (**b**) Polar diagram of the percentage increase of *I*_*0*_, %(I_0_–I_0,R_)/I_0,R_. for different spatial orientations of the input pulse. (**c**) Polar diagram of the relaxation time *t*_*R*_ of the depolarization drain current for different spatial orientations of the input pulse.

Spatial patterns of input voltage can be created by superimposing gate pulses at more than one (x, y) electrode. As shown in Fig. 3a, a spatial pulse of variable orientation is reproduced by applying voltage pulses (V_P_ = 0.3 V, t_P_ = 50 ms) concurrently at 3 different gate electrodes. For example, an orientation of 0° can be defined by applying pulses at the (x = 1–3, y = 2) electrodes, while orientation of 45° can be defined by applying pulses at the (x = 1–3, y = x) electrodes. Spatial voltage orientations from 0° to 180° with a step of 45° can be produced with an amplitude *I*_*0*_ defined for each orientation (for source-drain voltage V_DS_ = −0.2 V). The results are presented in Fig. 3b as a polar diagram of the increased percentage of *I*_*0*_ with respect to the lowest reference amplitude *I*_*0,R*_ (at 0° or 180°). The output amplitude *I*_*0*_ depends on the orientation of the spatial pulse pattern, with an increase of almost 60% in respect to *I*_*0,R*_ for the orientation of 45°. The spatial orientation of the input pulse is “encoded” in the form of a single output *I*_*0*_ response of the multi-gated OECT. This represents an analogy with the visual system, where a receptive field of the thalamus is projected onto a single visual cortex cell. The polar diagram of *I*_*0*_ exhibits an orientation selectivity towards the drain electrode, i.e., 45°. This selectivity towards 45° is attributed to a superimposed spatial pulse that includes the closest to the drain, gate electrode (x = 1, y = 1), which produces the maximum *I*_*0*_ amplitude since it defines the lowest resistance *R*_*E*_ (also refer to the experimental *I*_*0*_ spatial mapping of Fig. 2a or the corresponding theoretical mapping of Supplementary Fig. S3d).

The measured time *t*_*R*_ for the depolarization response of the drain current *I*_*D*_ is also calculated for the various spatial orientations of the input voltage, and the results are presented in Fig. 3c as a polar diagram of *t*_*R*_. Similarly, relaxation time selectivity towards 45° is also evident on the polar diagram of *t*_*R*_. Orientation of 45° produces the lowest *t*_*R*_ value, that is almost 50% lower than the other orientations. Again the spatial pulse of 45° includes the closest to the drain, gate electrode (x = 1, y = 1), and therefore results in the lowest relaxation time *t*_*R*_. It should be mentioned that spatial configurations other than 45°, such as (x = 1–3, y = 1) or (x = 1, y = 1–3), also result in maximum *I*_*0*_ and minimum *t*_*R*_, but these configurations are not used here for demonstration because they do not define any spatial orientation of the input pulse in respect to the central gate with coordinates (x = 2, y = 2).

## Discussion

We have demonstrated a sensing phenomenon analogous to orientation selectivity from the thalamus to the visual cortex with OECTs and electric stimuli as an input. It should be mentioned that cortical orientation maps already exist at the very early postnatal period, even in the absence of visual inputs, and are later strengthened by visual stimuli^33^,^34^. This work represents an ionic analogue of previsual orientation selectivity in a single OECT device. This approach differs from the existing approaches to orientation selectivity without an optical stimulus, in which grids of memristive devices^14^, or arrays of floating gate transistors are used^15^. In these works, orientation functionality is constructed by mapping the grids or arrays of several devices (e.g., mapping of resistance change in a memristive grid). A more complete, yet simplified model of orientation selectivity could be produced in future devices by integrating photodiodes at the gate electrodes in order to add spatially selective optical sensitivity. Orientation selectivity would be tuned by increasing the distance of the (x, y) gates from the PEDOT:PSS channel, or by using a channel with smaller dimensions. For example, the gates appear as point electrodes for large gate-channel distances and spatial mapping of *I*_*0*_ becomes more homogeneous. Moreover, two channels might also be placed perpendicular to each other, and would therefore be selective to different orientations of the spatial pulses of the (x, y) gate electrodes. This spatially-correlated function would have many potential applications, including the detection of ionic moving fronts and oriented ionic fluxes^35^, or spatially mapping ionic concentrations such as pH gradients^36^.

## Summary

In conclusion, a spatially-correlated function that was inspired by the visual system was reproduced in a single multi-gated OECT device. Gating the device in a global electrolyte permits a “soft-wired” and weighted connection of a single channel with spatially distributed multiple gate inputs and the device exhibits orientation selectivity with respect to spatially oriented voltage inputs. These results pave the way for organic bioelectronic applications with spatially resolved functionalities in processing and sensing.

## Methods

### Device fabrication

The devices were made using standard microfabrication techniques. As substrates, 26 mm × 76 mm glass slides were used. The contact lines were defined by evaporating a 10 nm Cr and a 100 nm Au layer on top of pre-patterned photoresist and a subsequent soaking in an acetone/isopropanol bath to remove the excess material. The Cr layer is needed to enhance surface adhesion between glass and Au. To protect the contact lines from the electrolyte, two parylene C layers were deposited on top, with 2 μm thickness each. The first was deposited on a surface treated with silane to enhance adhesion with the substrate and a thin 2% soap solution layer was spin-coated before the second deposition. This allows to peel-off the upper layer, thereby defining the device active area. The polymer used in this communication was PEDOT:PSS [Clevios PH 1000 from Heraeus Holding GmbH, with 5 wt% ethylene glycol, 0.1 wt% dodecyl benzene sulfonic acid and 1 wt% of (3-glycidyloxypropyl)trimethoxysilane)]. It was used both as the channel and as the gate. The desired film thickness (~500 nm) was obtained by three subsequent spin-coating steps with varying rotation speeds of 1500, 650, and 650 rpm respectively. A second photolithographic step was used to pattern these features. PEDOT:PSS spin-coating was followed by a hard**-**bake step at 140 °C for 60 min. The squared gate electrodes had an area of 3000 × 3000 μm^2^. Metal interconnects, insulated with parylene C, were used to make electrical contact. For detailed gate-channel distance mapping, refer to Figure S3a of the Supplementary Information section.

### Device characterization

The transistor was gated with (x, y) lateral Au electrodes covered with PEDOT:PSS, and aqueous NaCl electrolyte (100 mM) in deionized water. All measurements were recorded after cycling the OECTs (repetitive 0.3/−0.3 V cycles at the gate for 10 sec each) in order to obtain reproducible behavior. Each current amplitude *I*_*0*_ was defined for pulsing each gate with t_P_ = 50 ms and T_P_ = 10 s (using the average of 5 pulses) and recording the drain current. The amplitude *I*_*0*_ was quite stable for 5 pulses. When the devices are operated in normal conditions (using V_DS_ < 0.6 V and V_P_ < 0.6 V), they are quite stable for at least a six month period. OECT dimensions (PEDOT:PSS channel width, length and gate electrode) were determined with optical microscopy. The thickness of the PEDOT:PSS film was determined with profilometry. The drain current was measured using a National Instrument PXIe-1062Q system. The OECT was biased with a PXIe-4145 source measure unit (SMU) that was simultaneously recording drain current with a sampling rate of 1kHz. Gate voltages were generated by a National Instrument USB-6259. Both gate voltages and drain current measurements were internally triggered by the PXIe system. The acquisition system was monitored by custom-made LabVIEW software.

## Additional Information

**How to cite this article**: Gkoupidenis, P. *et al.* Orientation selectivity in a multi-gated organic electrochemical transistor. *Sci. Rep.* **6**, 27007; doi: 10.1038/srep27007 (2016).

## Supplementary Material

Supplementary Information
