# Supplementary material for: Orientation selectivity in a multi-gated organic electrochemical transistor
Source: Sci Rep. 2016 Jun 1;6:27007. doi: 10.1038/srep27007 (PMC4887893; doi:10.1038/srep27007)
Supplement: Supplementary Information [file srep27007-s1.doc]

**Supplementary information**

Orientation selectivity in a multi-gated organic electrochemical transistor

**Paschalis Gkoupidenis^1^, Dimitrios A. Koutsouras^1^, Thomas Lonjaret^1, 2^, Jessamyn A. Fairfield^3^, and George G. Malliaras^1^,***

^1^Department of Bioelectronics, Ecole Nationale Supérieure des Mines, CMP-EMSE, MOC, 13541 Gardanne, France

^2^MicroVitae Technologies, Hôtel Technologique, Europarc Sainte Victoire Bât 6, Route de Valbrillant, 13590 Meyreuil, France

^3^School of Chemistry and CRANN Institute, Trinity College Dublin, Dublin 2, Ireland

* [malliaras@emse.fr](mailto:malliaras@emse.fr)

**OECT’s time domain current response**

The equivalent circuit that describes the behaviour between the gate and the channel of the OECT consists of a resistor in series with a RC element,^1^ and is depicted in Fig. S1. *R_E_* corresponds to the electrolyte resistance and *R_P_*, *C_P_* to the PEDOT:PSS resistance and capacitance respectively. *I_E_(t)*, *I_P_(t)* and *I_C_(t)* are the time dependent currents of *R_E_*, *R_P_* and *C_P_* respectively. Then the overall current *I_E_(t)* is:

$I_{E}\left(t\right)=I_{P}\left(t\right)+I_{C}\left(t\right)$ (S1)

and the overall voltage of the equivalent circuit is V = V_E_(t) + V_C_(t), or

$V=I_{E}\left(t\right)R_{E}+I_{P}\left(t\right)R_{P}$ (S2)

The voltage across the parallel *R_P_C_P_* element can be written as:

$I_{C}\left(t\right)=C_{P}\frac{dV_{C}\left(t\right)}{dt}\Rightarrow V_{C}\left(t\right)=\frac{1}{C_{P}}\int_{0}^{t} I_{C}\left(t\right)dt$ (S3)

$I_{P}\left(t\right)R_{P}=V_{C}\left(t\right)\Rightarrow I_{P}\left(t\right)R_{P}=\frac{1}{C_{P}}\int_{0}^{t} I_{C}\left(t\right)dt$ (S4)

The Laplace transform of equation S4 is:

$I_{P}\left(s\right)=\frac{1}{R_{P}C_{P}}\cdot\frac{I_{C}\left(s\right)}{s}$ (S5)

From equation S1 and S5:

$I{}_{E}\left(s\right)=I_{C}\left(s\right)+\frac{1}{R_{P}C_{P}}\cdot\frac{I_{C}\left(s\right)}{s}\Rightarrow I_{E}(s)=\frac{1+R_{P}C_{P}s}{R_{P}C_{P}s}I_{C}\left(s\right)$ (S6)

By substituting equation S5 in the Laplace transform of equation S2:

$\frac{V}{s}=I_{E}\left(s\right)R_{E}+I_{P}\left(s\right)R_{P}\Rightarrow\frac{V}{s}=I_{E}\left(s\right)R_{E}+\frac{1}{R_{P}C_{P}}\cdot\frac{I_{C}\left(s\right)}{s}R_{P}$ (S7)

By substituting equation S6 in S7, the current of *C_P_*, *I_C_* can be expressed as:

$I_{C}\left(s\right)=\frac{1}{R_{E}}\frac{1}{\frac{R_{E}R_{P}}{R_{E}R_{P}C_{P}}+s}V$ (S8)

From the inverse Laplace transform of the equation S8, the time domain current *I_C_(t)* is written as:

$I_{C}\left(t\right)=\frac{V}{R_{E}}\exp\left(-\frac{R_{E}+R_{P}}{R_{E}R_{P}C_{P}}t\right)$ (S9)

From equation S4, the time domain current *I_P_(t)* is written as:

$$I_{P}\left(t\right)=\frac{1}{R_{P}C_{P}}\int_{0}^{t} I_{C}\left(t\right)dt=\frac{1}{R_{P}C_{P}}\frac{V}{R_{E}}\left[\int_{0}^{t} \exp\left(-\frac{R_{E}+R_{P}}{R_{E}R_{P}C_{P}}t\right)dt\right]$$

$I_{P}\left(t\right)=\frac{V}{R_{E}+R_{P}}\left[1-\exp\left(-\frac{R_{E}+R_{P}}{R_{E}R_{P}C_{P}}t\right)\right]$ (S10)

Finally, the overall time domain current *I_E_(t)* can be written as:

$I_{E}\left(t\right)=\frac{V}{R_{E}+R_{P}}\left[1-\exp\left(-\frac{R_{E}+R_{P}}{R_{E}R_{P}C_{P}}t\right)\right]+\frac{V}{R_{E}}\exp\left(-\frac{R_{E}+R_{P}}{R_{E}R_{P}C_{P}}t\right)$ (S11)

From equation S11, it comes out that the steady-state current for long polarization times (t → ∞) is:

$I_{E}\left(t\to\infty\right)=\frac{V}{R_{E}+R_{P}}$ (S12)

and the amplitude of the current *I_0_* at the initial moment of polarization (t → 0) is:

$I_{0}=I_{E}\left(t\to0\right)=\frac{V}{R_{E}}$ (S13)

The current amplitude *I_0_*, that is defined experimentally as the difference between the peak of the drain current and the steady-state response (see also Fig. 1), expresses qualitatively the gate current thought the electrolyte.

**Definition of the effective gate – PEDOT:PSS channel distance *d***

Figure S2 shows the experimental current amplitude *I_0_* as recorded for pulsing the (x = 1 - 3, y = 1) gate electrodes across the x direction (for V_P_ = 0.3 V, t_P_ = 50 msec, V_DS_ = -0.2 V). The amplitude *I_0_* decreases from the (x = 1, y = 1) to the (x = 3, y = 1) electrode. From the time domain response of the equivalent circuit of Fig. S1, the amplitude *I_0_* (for t → 0) can be expressed as I_0_ = V / R_E_ (equation S13). As a first order approximation, *R_E_* can be expressed as:

$R_{E}=\rho_{E}\frac{d}{A}$ (S14)

where *ρ_Ε_* is electrolyte resistivity, *A* is the electrode area and *d* is effective distance between the PEDOT:PSS channel and the center of the (x, y) gate electrode (for x, y = 1 - 3). It should be mentioned that in IZO-based synaptic transistors the output current amplitude was recently reported as a power law function of the distance *d*,^2^ but here for the sake of simplicity equation S14 is used. The effective distance *d* can be defined either from the gate to the PEDOT:PSS channel or to the drain electrode (see also simulation of *I_0_* mapping in Fig. S3a and S3b). From equation S13 and S14, it comes out that the *ρ_E_* is inversely proportional to the slope of the A_0_ – 1 / d diagram of Fig. S2, or ρ_E_ ~ 1 / slope. When *d* is defined as the distance from the channel, this leads to an electrolyte resistivity of ρ_E_ ~ 0. If *d* is defined as the distance from the drain electrode, this leads to ρ_E_ > 0. From Fig. S2 it can be referred that the decrease of *I_0_* across the x direction (or the calculation of the electrolyte resistivity *ρ_E_*) can be qualitatively explained by defining *d* rather from the gate to the drain electrode, than from the gate to the channel.

**Spatial mapping simulation of current amplitude *I_0_* in a multi-gated OECT**

Equations S13 and S14 express an amplitude of *I_0_* that depends (decreases) on the distance from the channel *d*. Here, the distance *d* is defined again by two ways; either from the PEDOT:PSS channel or the drain electrode. The spatial mapping of distance from the channel (in μm) is depicted in Fig. S3a. In Fig. S3c the normalized current amplitude I_0_/I_0max_ mapping is calculated according to Equations S13 and S14. As shown in Fig. S3c, the definition of *d* as the distance from the gate electrode to the channel, is insufficient to capture the experimental directivity towards the drain electrode (see also Fig. 2 and Fig. 3). In this case, the output amplitude *I_0_* is homogeneous across the x direction (e. g., for (x = 1 - 3, y = 1). Similarly, in Fig. S3b and S3d, the spatial mapping of distance *d* from the drain and the normalized amplitude I_0_/I_0max_ mapping is also depicted. The experimental orientation selectivity behavior is quantitatively captured when *d* is expressed as the distance from the gate to the drain electrode. The highest *I_0_* amplitude corresponds to the closest gate – drain distance, i.e. for the (x = 1, y = 1) electrode, while the lowest amplitude corresponds to the more distant gate (x = 3, y = 3), giving thus rise to an orientation selectivity behavior towards 45° (see also Fig. 3b).

**References**

^1^Rivnay, J. *et al.* *High-performance transistors for bioelectronics through tuning of channel thickness*. Vol. 1 (2015).

^2^Wan, C., Wu, G., Guo, L., Zhu, L. & Wan, Q. *e-print arXiv:1301.2052*.


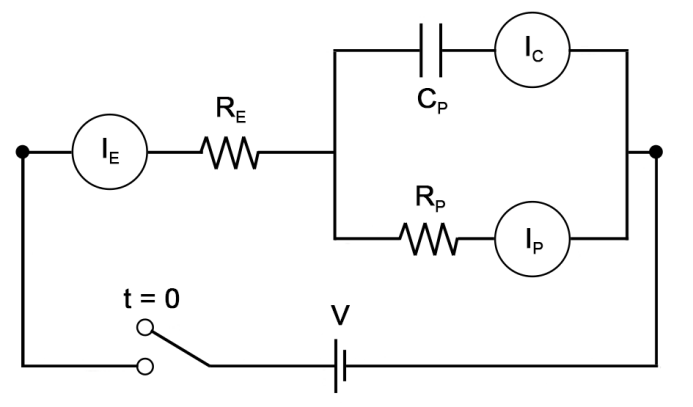


**Figure S1. Equivalent circuit that describes the behaviour between the gate and the channel of the OECT.**


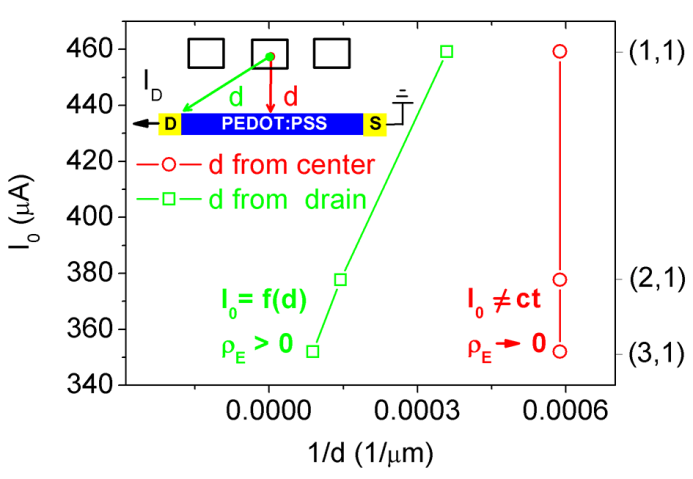


**Figure S2. Amplitude of the drain current *I_0_* vs the inverse of the distance *d*.** Amplitude of drain current *I_0_* when pulsing the (x = 1 - 3, y = 1) gate electrodes with a voltage amplitude V_P_ = 0.3 V and time width t_P_ = 50 msec as a function of the inverse effective distance of the (x, y = 1) gate to the PEDOT:PSS channel, 1 / d. The effective distance *d* is defined by two ways, either from the channel or from the drain electrode.


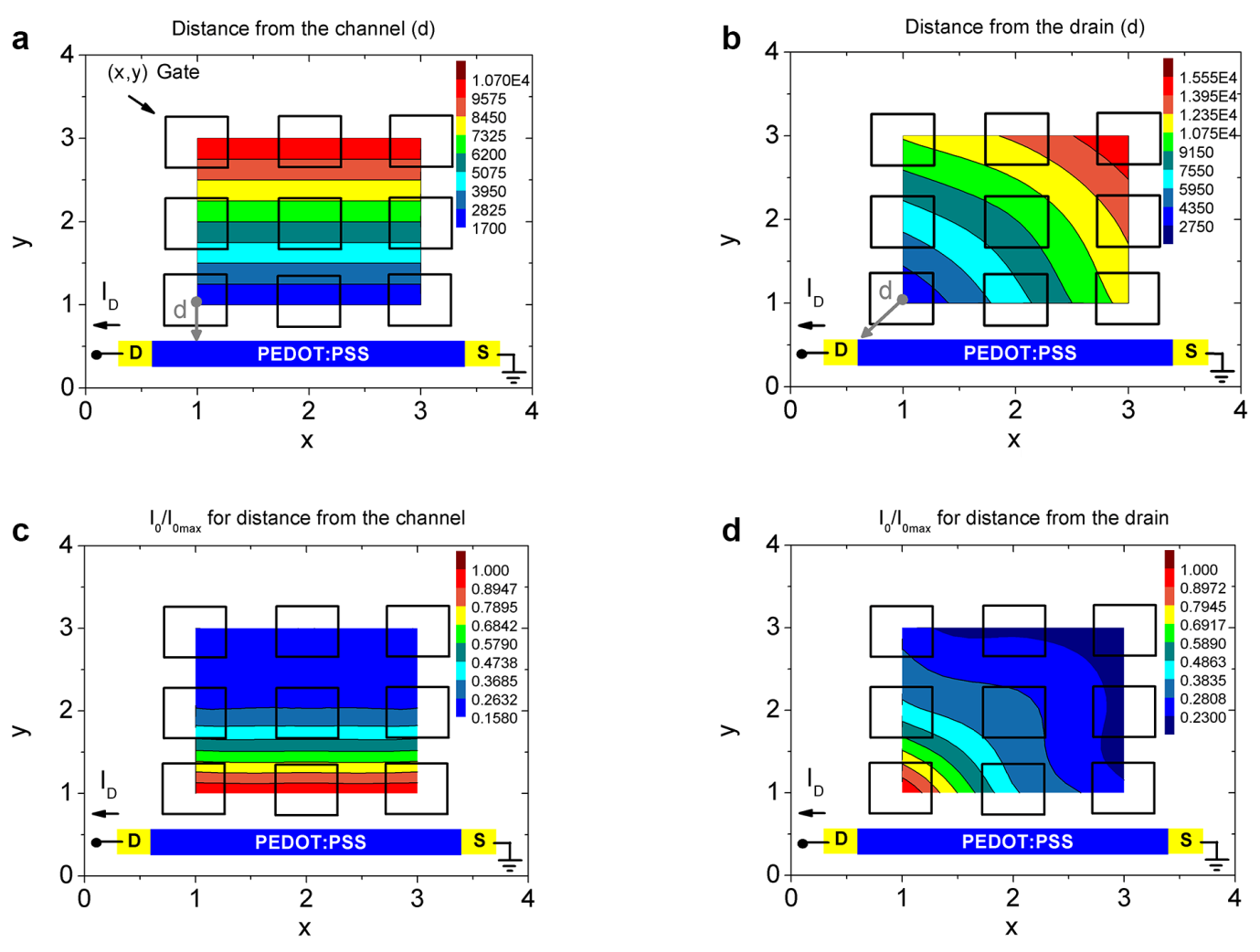


**Figure S3. Spatial mapping simulation.** Spatial mapping simulation of the effective distance *d* (in μm): (**a**) defined as the distance of the (x, y) gate to the PEDOT:PSS channel, (**b**) defined as the distance of the (x, y) gate to the drain electrode. Spatial mapping of the normalized drain current amplitude I_0_ / I_0max_ for *d*: (**c**) defined as the distance of the (x, y) gate electrode to the PEDOT:PSS channel, (**d**) defined as the distance of the (x, y) gate to the drain electrode.
